# Loss of physical contact in space alters the dopamine system in *C. elegans*

**DOI:** 10.1016/j.isci.2022.103762

**Published:** 2022-01-11

**Authors:** Surabhi Sudevan, Kasumi Muto, Nahoko Higashitani, Toko Hashizume, Akira Higashibata, Rebecca A. Ellwood, Colleen S. Deane, Mizanur Rahman, Siva A. Vanapalli, Timothy Etheridge, Nathaniel J. Szewczyk, Atsushi Higashitani

**Affiliations:** 1 Graduate School of Life Sciences, Tohoku University, 2-1-1 Katahira, Aoba-ku, Sendai, Miyagi 980-8577, Japan; 2 Medical Research Council (MRC) Versus Arthritis Centre for Musculoskeletal Ageing Research, Royal Derby Hospital, University of Nottingham, Derby, UK; 3 Musculoskeletal Conditions, National Institute for Health Research Nottingham Biomedical Research Centre, Derby, UK; 4 Advanced Engineering Services Co. Ltd, Tsukuba Mitsui Building7F,1-6-1 Takezono, Tsukuba, Ibaraki 305-0032, Japan; 5 Human Spaceflight Technology Directorate, Japan Aerospace Exploration Agency, 2-1-1 Sengen, Tsukuba, Ibaraki 305-8505, Japan; 6 Department of Sport and Health Sciences, College of Life and Environmental Sciences, University of Exeter, St. Luke's Campus, Exeter, UK; 7 Living Systems Institute, University of Exeter, StockerRoad, Exeter, UK; 8 Department of Chemical Engineering, Texas Tech University, Lubbock, TX, USA; 9 Ohio Musculoskeletal and Neurologic Institute, Ohio University, Athens, OH, USA; 10 Department of Biomedical Sciences, Heritage College of Osteopathic Medicine, Ohio University, Athens, OH, USA

**Keywords:** Space medicine, Aerospace Engineering

## Abstract

Progressive neuromuscular decline in microgravity is a prominent health concern preventing interplanetary human habitation. We establish functional dopamine-mediated impairments as a consistent feature across multiple spaceflight exposures and during simulated microgravity in *C. elegans*. Animals grown continuously in these conditions display reduced movement and body length. Loss of mechanical contact stimuli in microgravity elicits decreased endogenous dopamine and *comt-4* (catechol-O-methyl transferase) expression levels. The application of exogenous dopamine reverses the movement and body length defects caused by simulated microgravity. In addition, increased physical contact made *comt-4* and dopamine levels rise. It also increased muscular cytoplasmic Ca^2+^ firing. In *dop-3* (D2-like receptor) mutants, neither decrease in movement nor in body length were observed during simulated microgravity growth. These results strongly suggest that targeting the dopamine system through manipulation of the external environment (contact stimuli) prevents muscular changes and is a realistic and viable treatment strategy to promote safe human deep-space travel.

## Highlights

• Dopamine levels, movement and body are reduced in *C. elegans* grown under microgravity
• Loss of mechanical contact in microgravity activates a D2-like dopamine receptor
• Reinstating physical contact stimulation improves these reductions

## Graphical abstract

**Figure:**
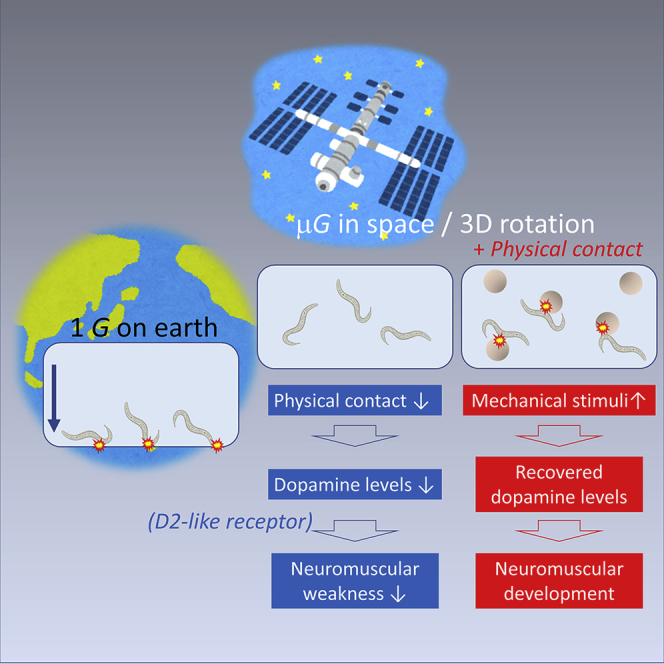

## Introduction

Over almost 60 years, hundreds of humans have flown into space, spending up to one year living on the International Space Station (ISS). Unlike living on Earth, spaceflight presents a multitude of environmental stressors such as microgravity (μG) and cosmic radiation exposure. The direct consequences of these unique features of spaceflight upon health are widespread, including declines in the skeletal, neuromuscular, sensory, vestibular, and cardiovascular systems (Furukawa et al., 2021; Gallo et al., 2020; Morita et al., 2016; Nicogossian et al., 2016). Achieving the great human ambition of interplanetary habitation requires effective countermeasures against these μG health concerns which, at present, are lacking.

Given the significant costs and practical limitations of studying astronauts, model organisms have also been employed. They have been found to demonstrate highly comparable molecular and physiological maladaptations to spaceflight that are parallel to those observed in people. For example, we have shown the nematode *Caenorhabditis elegans* grown in μG displays reduced muscle cytoskeletal proteins, mitochondrial metabolic enzymes (Higashibata et al., 2016), and DAF-2/insulin/IGF-1 signaling (Willis et al., 2020). This molecular profile translates to impaired muscular performance and body length (Higashibata et al., 2016), paralleling functional changes reported in astronauts (Fitts et al., 2010; Nicogossian et al., 2016). However, it is unclear whether gravity-related health decline is a direct result of μG or the indirect effects of floating. For example*, C. elegans* are small (∼1 mm long, weighing ∼1 μg) and experience buoyancy during liquid culture in μG, resulting in almost complete loss of contact stimuli. Conversely, in the 1G Earth environment, nematodes settle in liquid and experience contact stimuli with each swimming movement. Altered mechanosensory signaling might therefore contribute to impaired neuromuscular health in-flight.

The neurotransmitter dopamine (DA) is a well-established modulator of mechanosensory plasticity in *C. elegans* (Allen et al., 2011; Chase et al., 2004; Ezak and Ferkey, 2010; Han et al., 2017; Kindt et al., 2006; Li et al., 2006; Sanyal et al., 2004; Sawin et al., 2000; Xu et al., 2021). *C. elegans* DA neurons are sensory neurons that are morphologically similar to the sensory neurons present in the vertebrate inner ear (Li et al., 2006). These *C. elegans* neurons have been reported to sense the mechanical properties of contact with and texture of the surface upon which the worm moves. For example, as worms enter a patch of food, the change in mechanical stimuli alters dopamine signaling to slow animals down, a behavior known as the basal slowing response. Similarly, in the absence of bacteria, the body bends more often (Chase et al., 2004; Li et al., 2006; Sawin et al., 2000). In addition to modulating these behaviors, touch-dependent dopamine signaling regulates spatial pattern selectivity (Han et al., 2017). While there are at least 5 dopamine receptors in *C. elegans*, the D1-like receptor (D1R) DOP-1 and the D2R DOP-3, expressed in ventral cord motor neurons which innervate the body wall muscles, control locomotion (Han et al., 2017; Sawin et al., 2000). DOP-1 and DOP-3 antagonize each other in the same neuronal cells (Allen et al., 2011; Chase et al., 2004) with DOP-3 controlling the slowing response and contributing to spatial pattern selectivity, regulation of olfactory sensitivity, and the swimming behavior (Allen et al., 2011; Chase et al., 2004; Ezak and Ferkey, 2010; Han et al., 2017; Xu et al., 2021). DA also regulates *C. elegans* body length (Nagashima et al., 2016) and synapses onto motoneurons, plausibly linking μG induced decreases in body length and impaired muscular performance (Higashibata et al., 2016).

Furthermore, as with vertebrates, DA regulates learning, decision making, and neuromuscular gait transitions (Kimura et al., 2010; Vidal-Gadea et al., 2011; Voglis and Tavernarakis, 2008). This critical role of DA is illustrated by the onset of dysfunctional motor output and Parkinson disease upon loss of striatal DA in humans (Ammann et al., 2020; Charvin et al., 2018; Fahn 2008; Fearnley and Lees, 1991; Kordower et al., 2016). Interestingly, a one-month Russian biosatellite BION-M1 spaceflight study of mice reported for the first time that the expression of certain genes involved in dopamine synthesis and degradation were reduced in the brain (Popova et al., 2015). Importantly, DA agonists are a mainstay of Parkinson disease therapy (Iarkov et al., 2020) and improve skeletal muscle mass, tone, and function during muscle atrophying conditions (Reichart et al., 2011; Schwarz and Peever 2011). Establishing the role of mechanosensory-related DA signaling in μG would thus promote clinically viable therapeutics for neuromuscular health maintenance in spaceflight.

## Results and discussion

### Spaceflight and simulated μG result in decreased *comt-4* expression and endogenous dopamine in *C. elegans*

**Figure 1:**
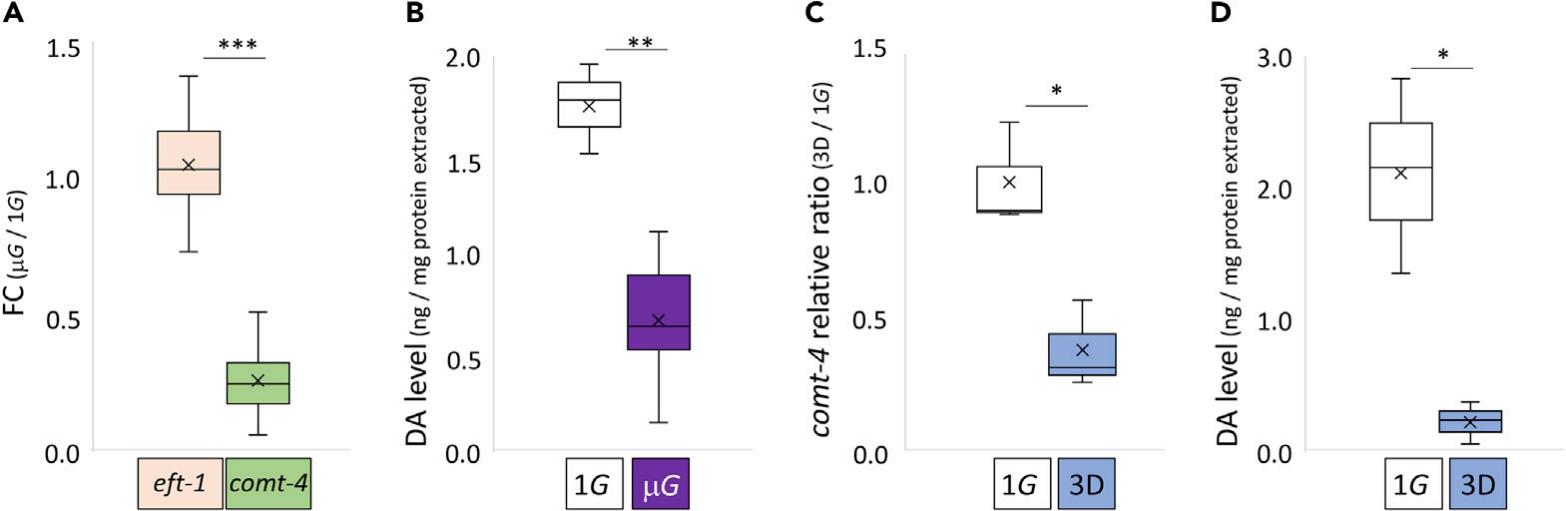
Decreased *comt-4* expression and endogenous dopamine levels of *C. elegans* grown under space microgravity and artificial microgravity with 3D clinorotation (A) Changes in gene expression (fold change values with DNA microarray analyses) of *eft-1* and *comt-4* across all 15 independent specimens grown under microgravity and artificial 1G conditions or ground control (detailed in Figure S1B). (B) Endogenous DA levels in wild-type N2 adult hermaphrodites grown on the ground (1G) and space microgravity (μG). (C) Expression levels of *comt-4* were analyzed by real-time RT-PCR with *eft-2* internal standard in N2 adults grown on the ground (1G) and simulated μG with 3D clinorotation (3D). (D) Endogenous DA levels in wild-type N2 adults grown on the ground (1G) and simulated μG (3D). Data are shown as box and whiskers to indicate median and SD. Statistical analysis was performed in each condition using Student’s *t* test. *∗*p< 0.05, *∗∗*p< 0.01, and *∗∗∗*p< 0.001.

*C. elegans* grown under μG onboard the ISS over three separate ISS flight experiments (total of 15 populations) consistently display decreased *comt-4* gene expression, a catechol-O-methyltransferase DA degradation enzyme (Rodríguez-Ramos et al., 2017), versus 1G centrifuge or ground control (Figures 1 and S1). In the 2009 CERISE experiment to evaluate RNAi activity in space (Etheridge et al., 2011; Higashibata et al., 2016; Higashitani et al., 2009), *comt-4* expression was reduced to approximately 30% in both 1^st^ and 2^nd^ generation N2 wild-type adults grown under microgravity compared to those grown in 1G centrifuge onboard ISS (Figure S1). In the 2015 EPIGENETICS experiment to monitor epigenetic changes in space (Higashitani et al., 2021), N2 wild-type and histone deacetylase mutants, *hda-4* and *sir-2.1* were cultured over four generations on the ISS microgravity and artificial 1G centrifuge, all strains displayed decreased *comt-4* expression in all four generations under microgravity compared with 1G condition (Figure S1). Lastly, in the 2018 Molecular Muscle Experiment (Pollard et al., 2020), PD55 (worms expressing *lacZ* in muscle) also displayed decreased *comt-4* compared with ground controls (Figure S1). In contrast, expression of DA receptor genes, *dop-1*, *-2*, *-3*, *-4*, and *-5* did not change significantly (Figure S1).

Given the role of COMT-4 in DA degradation (Rodríguez-Ramos et al., 2017), we measured endogenous DA levels of wild-type adults grown during our recent ISS μG experiment (Pollard et al., 2020). We found DA levels reduced by more than half versus ground controls (Figure 1). As this observation suggested *comt-4* expression might be under the control of endogenous DA levels, we also tested the effect of exogenous DA application on Earth. We found increased expression of *comt-4* 24 h after exogenous DA application (using OP-50 NGM agar plate: Figure S2). Lastly, to determine if μG rather than some other element of spaceflight such as cosmic radiation was regulating DA signaling, we examined *comt-4* expression and endogenous DA levels during 3D clinorotation-simulated μG, both were reduced as predicted (Figure 1, Video S1).

**Figure 2:**
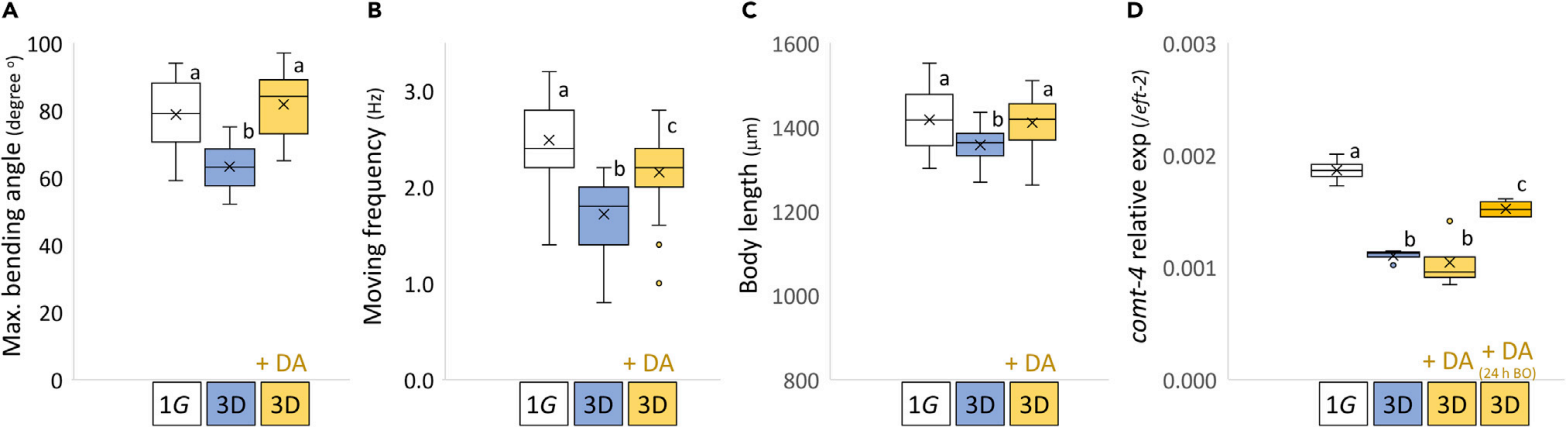
Supply of exogenous dopamine restored moving activity and physique loss under artificial microgravity (A–D) Maximal bending angle (n = 15 each), (B) moving frequency as thrashing rate (n = 30 per condition), (C) body length (n = 30 per condition), and (D) expression levels of *comt-4* were analyzed in wild-type N2 day 1 adult hermaphrodites grown on the ground (1G) and simulated μG (3D) with or without a final 50μM DA treatment (3,4-dihydroxyphenethylamine hydrochloride). DA was added at the start of culture at the L1 larval stage (+ DA) or in adult animals 24 h before observation (+DA (24 h BO) for only *comt-4* analysis). Data are shown as box and whiskers to indicate median and standard deviations. Statistical analysis was performed in each condition using one-way ANOVA followed by Tukey post hoc test. Different letters indicate statistically significant differences at p< 0.05.

**Figure 3:**
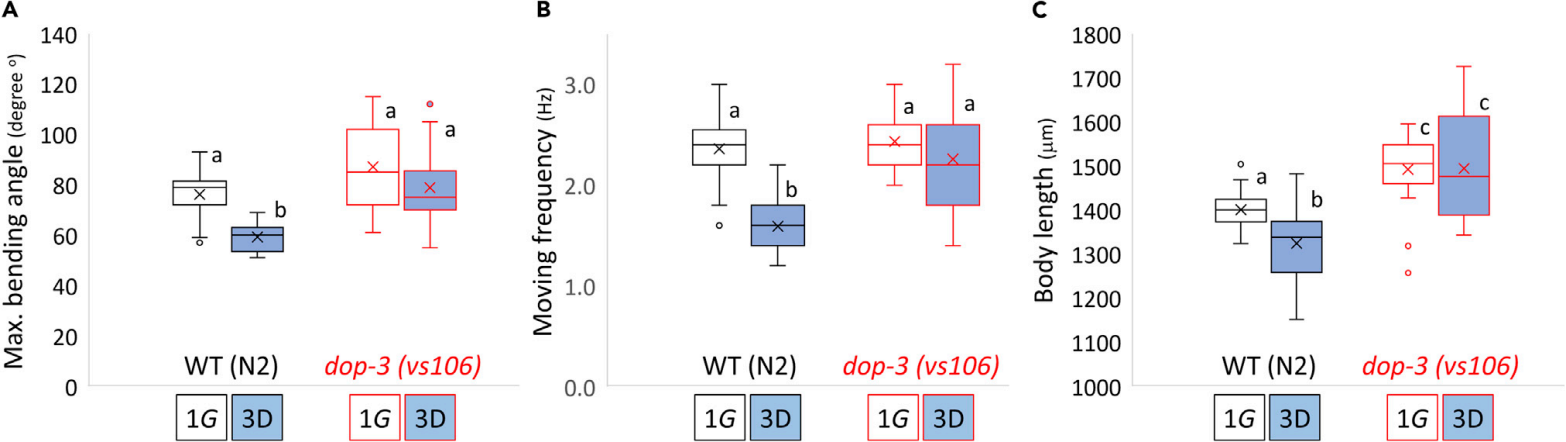
D2-like receptor dop-3 mutation restored moving activity and physique loss under artificial microgravity (A–C) Maximal bending angle (n = 15 per condition), (B) moving frequency as thrashing rate (n = 30 per condition), and (C) body length (n = 30 per condition) were measured in day 1 adults of N2 and *dop-3 (vs106)* deletion mutant cultured parallelly under normal gravity (1G) and simulated μG (3D) for 4 days. Data are shown as box and whiskers to indicate median and standard deviations. Statistical analysis was performed in each condition using one-way ANOVA followed by Tukey post hoc test. Different letters indicate statistically significant differences at p< 0.05.

**Figure 4:**
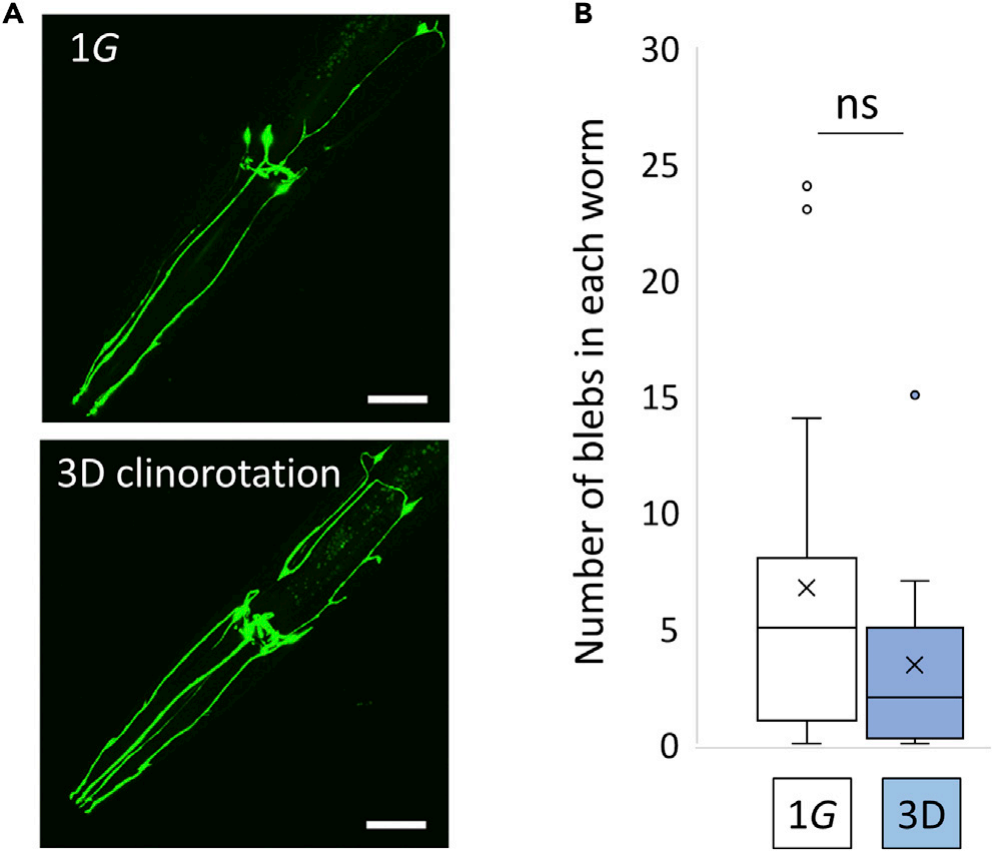
Simulated microgravity did not change DA neuron morphology (A) Day 4 adults *(vtIs1)* under 1G or 3D clinorotation. Scale bars: 20 μm. (B) DA neurodegeneration, as an increase in the number of blebs along dendrites in animals under 1G or 3D clinorotation. Data are shown as box and whiskers to indicate median and standard deviation. Statistical analysis was performed in each condition using Student’s *t* test. ns: not significant.

**Figure 5:**
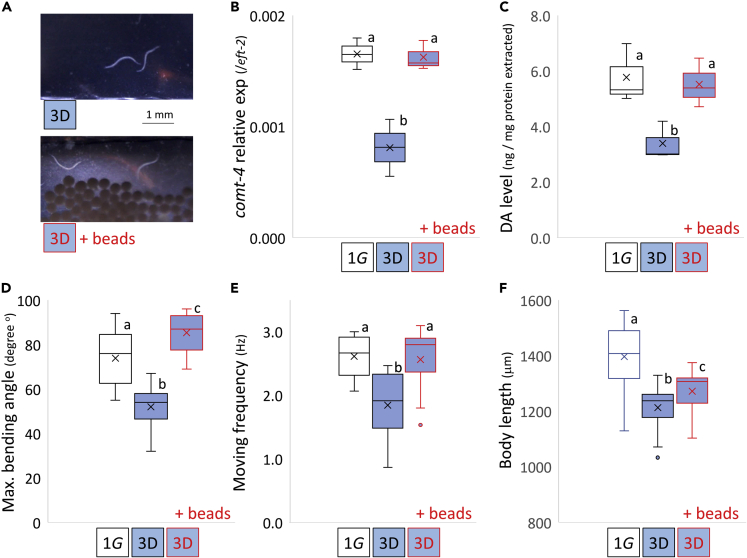
Increased contact stimuli with the supply of microbeads restored locomotion activity and loss of physique under artificial microgravity (A) D1 adults *(vtIs1)* grown in culture bags with or without microspheres under 3D clinorotation (Video S4). (B–F) Expression levels of *comt-4* by real-time RT-PCR with *eft-2* internal standard, (C) endogenous DA levels, (D) maximal bending angle (n = 15 per condition), (E) moving frequency as thrashing rate (n = 30 per condition), and (F) body length (n = 30 per condition) was measured in day 1 adults *(vtIs1)* parallelly cultured for 4 days under normal gravity (1G) and simulated μG (3D) in the absence or presence of microspheres (‘+ beads’). Data are shown as box and whiskers to indicate median and standard deviations. Statistical analysis was performed in each condition using one-way ANOVA followed by Tukey post hoc test. Different letters indicate statistically significant differences at p< 0.05.

**Figure 6:**
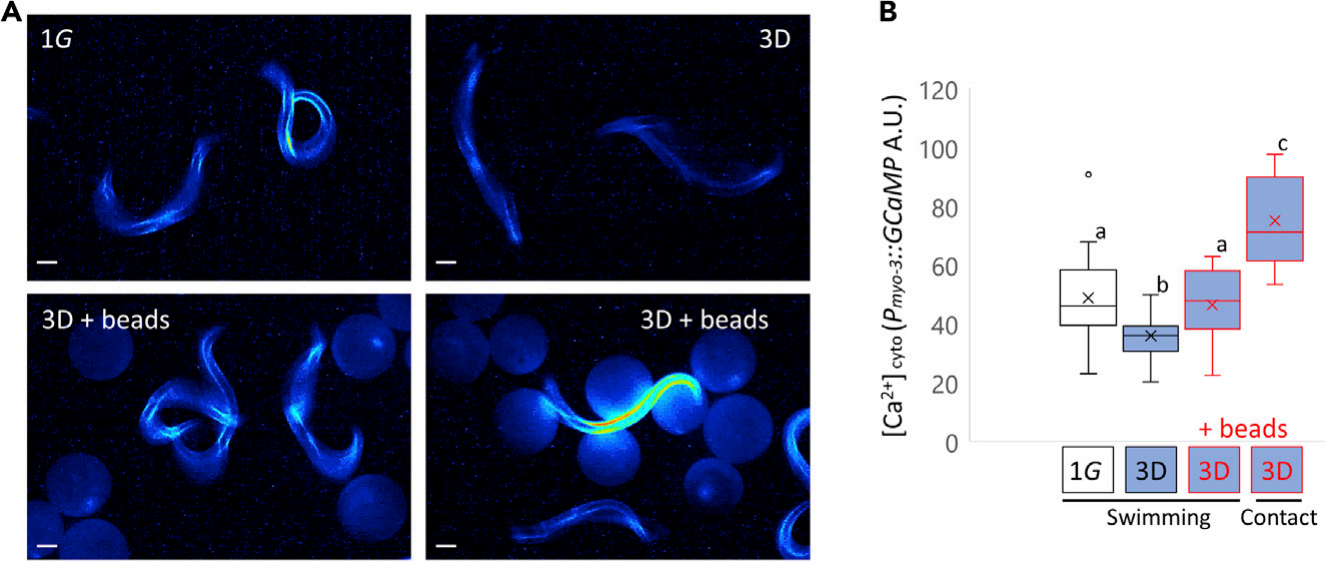
Contact stimulation increases muscle cytoplasmic Ca^2+^ firing (A) Changes in muscular cytoplasmic Ca^2+^ levels of day 1 adults (*goeIs3*: *Pmyo-3::GCaMP*) grown at 1G or μG (3D) for 4 days with or without microsphere beads (‘+ beads’) were captured under the same fluorescent intensities from video images (Video S5). GFP fluorescent signals were converted using image J royal color. Scale bars: 100 μm. (B) Maximal signal intensity of muscle cytoplasmic Ca^2 +^ levels of contract site under swimming behavior and contact beads (n = 16 animals per condition) were measured using image J software. Data are shown as box and whiskers to indicate median and standard deviation. Statistical analysis was performed in each condition using one-way ANOVA followed by Tukey post hoc test. Different letters indicate statistically significant differences at p< 0.05.

Video S1. Demonstration of the 3D clinostat used to simulate microgravity, related to Figures 1–6 and STAR Methods

### Exogenous dopamine reverses the movement and body length defects caused by μG

Previously, spaceflight has been shown to reduce movement rates, an established index of neuromuscular and overall animal health (Bansal et al., 2015), bending angles, and body length (Higashibata et al., 2016) (Figure S3). We confirmed these changes also occur in simulated μG on Earth using a 3D-clinostat (Video S1, Figure 2). Consistent with decreased DA levels causing these changes in μG, we found administration of 50 μM exogenous DA across developmental stages (from L1 larvae to D1 adults for 4 days) prevented movement abnormalities associated with 3D clinorotation μG (Figure 2, Video S2) and also prevented the decreased body length associated with simulated μG (Figure 2). Exogenous DA also resulted in increased expression of *comt-4* 24 h after application of exogenous DA in simulated μG in young adults, similar to results from the plate 1G study (Figures 2 and S2). However, in D1 adults treated with DA from the L1 larval stage, the expression levels of *comt-4* returned to untreated levels (Figure 2). Therefore, the increased expression of *comt-4* by exogenous DA treatment occurs as a relatively transient response, which may differ from the constant regulation of expression with changes in endogenous DA levels.

Video S2. Supply of exogenous dopamine restored moving activity and physique loss under artificial microgravity, related to Figure 2

We found that *C. elegans* swam much slower when grown in a microgravity environment. This appeared to be caused, in part, by low levels of DA because μG-cultured worms had lower levels of DA and exogenous treatment with DA restored swimming to normal speed. However, the decreased speed of μG-treated worms may need to be explained by factors beyond insufficient DA because a previous study found that decreasing DA by mutation (*cat-2*) or cell ablation caused worms to crawl faster than wildtype on seeded plates (Sawin et al., 2000). Factors to consider for future study include different methodologies for evaluation of locomotory activity, such as uniform and sufficient foods from liquid culture, maximum locomotory rates in swimming after gentle stimuli, and continuous growth and development in liquid with only swimming from L1 larvae to adults. Alternatively, reduced expression of mitochondrial enzymes as well as muscle proteins, changes in the expression of the BMP/TGF-β growth factor gene *dbl-1* and the epigenetic modification of some genes have been observed in our spaceflight samples (Higashibata et al., 2016; Higashitani et al., 2021). Thus, changes in DA levels integrated in the context of additional biological changes may also explain the differences. Future investigations of the effects of microgravity in space using strains with reduced dopamine levels, such as the *cat-2*mutant, is an important issue for the future.

### D2R DOP-3 mediates the effects of μG on movement and body length

Integration of DA signals to control movement occurs via *dop-1* and *dop-3.* We therefore investigated the μG-induced reductions in maximum bending angle and body length in the loss-of-function mutants of D1R *dop-1* (*vs100*) and D2R *dop-3 (vs106)*.While μG-induced reductions in bending angle and body length in D1R *dop-1* (*vs100*) mutants (Figure S4), μG failed to induce significant reductions in bending angle and body length in D2R *dop-3 (vs106)* mutants (Figure 3, Video S3). The inability of μG to induce changes in *dop-3* mutants suggest that DOP-3 and not DOP-1 is mediating the DA effect caused by exposure to μG. Vertebrate DA receptors have been reported to have significant differences in DA affinity between D1Rs and D2Rs (Richfield et al., 1989) with D1Rs displaying low affinity (high dissociation constant: $K_{d}^{\mathrm{D1}}$ = 1.6 μM) and D2Rs displaying high affinity (low dissociation constant: $K_{d}^{\mathrm{D12}}$ = 25nM). Thus, reduced DA levels in response to μG might be preferentially increasing inhibitory signals via high affinity D2Rs, while disproportionately lowering signals through low affinity D1Rs, to decrease movement and body length. Importantly, these inhibitory effects can be reversed by treatment with exogenous DA.

Video S3. D2-like receptor *dop-3* mutation restored moving activity, related to Figure 3

### Mechanical stimulation prevents μG-induced reduced DA signaling and movement decline

We also examined whether the decrease in DA caused by μG was associated with degenerated dopamine neurons. In *C. elegans*, DA neurons are visualized using green fluorescent protein regulated by the DAT (DopAmine Transporter) gene promoter (Masoudi et al., 2014), and these worms are utilized as a Parkinson disease model (Nass et al., 2002; Saha et al., 2015; Gaeta et al., 2019). Abnormal blebs along dendrites increase with neurotoxin treatment and GFP fluorescent levels decrease with age. Similar to wild-type animals, simulated μG caused a decrease in DA levels and neuromuscular activity in *Pdat-1::GFP* worms, but number of blebs along dendrites and GFP fluorescent levels did not change in D4 (day 4) adult (Figures 4 and 5). These results imply that DA synthesis and metabolism change in response to μG in the absence of gross DA neuron degeneration. However, because reduced body size and behavioral responses induced by removing animal–animal contact stimuli also lowers expression of touch neuron synaptic transmission signals (e.g., SNB-1, Rose et al., 2005), assessment of alternative neuronal structures/pathways in μG might warrant future investigation.

As DA neurons display well-established mechanosensitivity (Sanyal et al., 2004), we tested if increased contact stimulation during simulated μG prevented alterations in DA signaling in μG. Adult animals grown throughout development (Length at L1 larvae: approximately 400 μm and at adults: approximately 1,300 μm, with a length-width ratio of about 30:1) in μG and polyethylene microsphere beads (ɸ250–300 μm, 1.00 g/cc) with the same specific density as water displayed no decline of *comt-4* expression, DA levels or moving behaviors as compared with adults grown in the absence of the beads (Figure 5, Video S4). Thus, lack of physical contact stimulation during development in μG appears to underlie changes in DA-mediated maintenance of the neuromuscular system in response to μG.

Video S4. Increased contact stimuli with the supply of microbeads restored locomotion activity and loss of physique under artificial microgravity, related to Figure 5

To confirm increased contact stimuli improved neuromuscular function, we examined the effect of microsphere beads on cytoplasmic Ca^2+^ levels in muscle. Using a *Pmyo-3::GCaMP* transgene (*goeIs3* [*Pmyo-3::SL1::GCamP3.35::SL2::unc54 3′UTR + unc-119(+)*] *V*), we observed the Ca^2+^ levels during muscle contraction during the swimming behavior of D1 adults grown in simulated μG in the presence or absence of beads and also in the 1G environment (Figure 6, Video S5). The maximum Ca^2+^ levels were significantly reduced in adults grown in simulated μG compared to 1G. The addition of beads to μG-grown worms not only improved the maximum bending angles but also increased Ca^2 +^ levels. In addition, higher muscle cytoplasmic Ca^2 +^ levels were observed when worms were in direct contact with a bead (Figure 6). The provision of adequate physical contact stimulation therefore associates with improved calcium handling and, as a result, might promote improved neuromuscular activity during μG.

Video S5. Contact stimulation altered locomotory gait and increased Ca^2+^ firing in the muscles, related to Figure 6

### Conclusion

Here, we report that μG associated with spaceflight and 3D clinorotation causes decreased DA levels and reduced neuromuscular activity, which can be reversed via treatment with exogenous DA. Moreover, we establish a contact stimulus > DA > muscle regulatory axis that controls motor decline in μG. Restoration of this axis by the introduction of physical touch stimuli in animal culture environments is sufficient to improve health. This supports rodent data reporting reduced DA synthesis, Comt-degradation, and D1R genes' expression during spaceflight (Popova et al., 2015). Interestingly, the hindlimb unloading rodent model (a commonly employed spaceflight ground analog) displays oppositely increased expression of dopaminergic genes including Comt and D1R (Kulikova et al., 2017). While counterintuitive, hindlimb unloading imposes mechanical stress on the forelimbs and tail, likely inducing a global mechanosensory dopaminergic response. Combined, these data support a model whereby reduced contact stimuli, rather than unloading *per se*, reduces physiological DA to impair neuromuscular performance. Thus, because dopamine and its agonists have various side effects, suppressing the reduction of endogenous dopamine by mechanical stimulation may be an effective method for combating space-flight-induced neuromuscular atrophy.

## STAR★Methods

### Key resources table

**Table:** 

REAGENT or RESOURCE	SOURCE	IDENTIFIER
**Bacterial and virus strains**		

*Escherichia coli* OP-50	NemaMetrix Co. Ltd.	LabTIE OP-50 V.2

**Chemicals, peptides, and recombinant proteins**		

3.4-Dihydroxyphenethylamine Hydrochloride	FUJIFILM Wako Pure Chemical Corporation	4987481383029
polyethylene microsphere beads	Cospheric LLC	WPMS-1.00 ɸ250-300 μm (1.00 g / cc
DNase I (RNase-free)	Takara Bio	2270A
Western Lightning Plus-ECL chemifluorescence kit	PerkinElmer	NEL103001EA
TRI reagent	Molecular Research Center	TR 118200ML
PrimeScript RT Reagent Kit with gDNA Eraser	Takara Bio	RR047A
*C. elegans* Oligo Microarray 44 k version 2.0	Agilent Technologies	https://www.chem-agilent.com/contents.php?id=29452

**Critical commercial assays**		

Dopamine ELISA kit - Research	ImmuSmol	BA-E-5300

**Deposited data**		

*C. elegans* Microarray data (CERISE exp)	Higashibata et al., 2016	GEO: GSE71770
*C. elegans* Microarray data (EPIGENETICS exp)	Higashitani et al., 2021	GEO: GSE173985

**Experimental models: Organisms/strains**		

*C. elegans*: Strain: N2 (WT).	Caenorhabditis Genetics Center	WB Strain: 00000001
*C. elegans*: Strain LX703: *dop-3 (vs106)*.	Caenorhabditis Genetics Center	WB Strain: 00026374
*C. elegans*: Strain LX645: *dop-1 (vs100)*.	Caenorhabditis Genetics Center	WB Strain: 00026369
*C. elegans*: Strain TG2435: *vtIs1 [Pdat-1::GFP + rol-6]*.	Caenorhabditis Genetics Center	WB Strain: 00034694
*C. elegans*: Strain ATU2301: *goeIs3 [Pmyo-3::GCaMP3.35::unc-54-3'utr, unc-119(+)] aceIs1 [Pmyo-3::mitochondrial LAR-GECO + Pmyo2::RFP]*.	This paper	N/A

**Oligonucleotides**		

Forward Primer for *comt-4*: 5’ CGCTGCGATTCACGAGATG,	This paper	N/A
Reverse Primer for *comt-4*: 5’ GAAGCGCCGAGTAGGTACGAT	This paper	N/A
Forward Primer for *eef-2*: 5’ GACGCTATCCACAGAGGAGG,	This paper	N/A
Reverse Primer for *eef-2*: 5’ TTCCTGTGACCTGAGACTCC	This paper	N/A

**Recombinant DNA**		

transgene *aceIs1 [Pmyo-3::mitochondrial LAR-GECO + Pmyo2::RFP]*	This paper	N/A
transgene *goeIs3 [Pmyo-3::GCaMP3.35::unc-54-3'utr, unc-119(+)]*	Caenorhabditis Genetics Center	WBTransgene00018927
transgene *vtIs1 [Pdat-1::GFP + rol-6]*	Caenorhabditis Genetics Center	WBTransgene00004906

**Software and algorithms**		

CellSens imaging software	Olympus	CellSens Standard 2.2
ImageJ for fluorescent image analysis	NIH	https://imagej.nih.gov/ij/
one-way ANOVA followed by Tukey post hoc tests for statistics	RStudio Software	https://www.rstudio.com/products/rstudio/
Microsoft Excel 2019 for data presentation	Microsoft	https://www.microsoft.com/

### Resource availability

#### Lead contact

Further information and requests for resources and reagents should be directed to and will be fulfilled by the lead contact, Atsushi Higashitani (atsushi.higashitani.e7@tohoku.ac.jp)

#### Materials availability

The ATU2301 nematode strain constructed in this study is available from the authors in accordance with the Material Transfer Agreement.

### Experimental model and subject details

#### *Caenorhabditis elegans* strains and culture methods

Four strains of *C. elegans* were used in this study: N2 (WT strain), LX703 *dop-3 (vs106)*, TG2435 (*vtIs1* [*Pdat-1::GFP + rol-6*] and ATU2301 (*goeIs3* [*Pmyo-3::SL1::GCamP3.35::SL2::unc54 3'UTR + unc-119(+)*]; *aceIs1*[*Pmyo-3::mitochondrial LARGECO+Pmyo-2::RFP*]). To synchronize growth, approximately 50 adult hermaphrodites were transferred to a fresh NGM plate containing *E. coli* OP50 and cultured overnight at 20°C. Eggs laid were collected by flushing the adult animals with M9 buffer. After overnight incubation, L1 larvae were collected from the plate by using M9 buffer. Approximately 500 L1 larvae were cultured into 5 ml (35 mm x 60 mm polyethylene bag) of S liquid media containing 2 x freeze dried *E. coli* OP50 (NemaMetrix Co. Ltd.) at 20°C. Each bag was sandwiched with plastic mesh seats (50 mm x 75 mm (with 3 mm x 3 mm hole)) and setting into polycarbonate container (iPTEC ® secondary container PC-0.5, SANPLATEC corp.) with sandwiched with hole pushing contents (iP-TEC® secondary container private mesh cushion). The container was subjected to microgravity treatment using a 3D-clinostat (Portable Microgravity Simulator PMS-Ⅶ; Advanced Engineering Services Co., Ltd.,Video S1) in thermal incubator (LTI-700; EYELA Co. Ltd.) at 20°C for 4 days (D1 adult) or 7 days (D4 adult).

For exogenous dopamine application, 3.4-Dihydroxyphenethylamine Hydrochloride (dopamine hydrochloride, FUJIFILM) was supplemented into the culture bag starting from L1 larvae at a final concentration of 50 μM in 5 ml 2 x freeze dried OP50 medium. To increase contact stimuli, 0.5 g of polyethylene microsphere beads (WPMS-1.00 ɸ250-300 μm (1.00 g / cc), Cospheric LLC, Santa Barbara) was added into 5 ml medium.

### Method details

#### Spaceflight experiments

In this study, we used spaceflown *C. elegans* samples derived from our “CERISE”,“EPIGENETICS” and “MME” spaceflight experiments (Figure S1), the experimental protocols of which are fully described in (Etheridge et al., 2011; Higashibata et al., 2016; Higashitani et al., 2009, 2021; Pollard et al., 2020). All flight samples were frozen at -95°C in the ISS freezer MELFI, and returned to Earth under frozen conditions. The cells were thawed in each laboratory and the following gene expressions and dopamine levels were analyzed rapidly and quantitatively.

#### Expression analysis

Total RNA was isolated using a TRI Reagent (Molecular Research Center, Cincinnati) following the manufacturer’s protocol. The residual DNA was eliminated by treating with DNase I (Takara Bio, Shiga, Japan) during an isolation process of total RNA. The isolated total RNA was subjected to DNA microarray and real-time qPCR. The isolated RNA was analyzed on the *C. elegans* Oligo Microarray 44 k version 2.0 (Agilent Technologies, Santa Clara, CA, USA). Realtime quantitative reverse transcription polymerase chain reaction analysis was performed with the following primer sets: *comt-4*: fw 5’-CGC TGC GAT TCA CGA GAT G, rv 5’-GAA GCG CCG AGT AGG TAC GAT, *eef-2*: fw 5’-GAC GCT ATC CAC AGA GGA GG, and *eef-2*: rv 5’-TTC CTG TGA CCT GAG ACT CC.

#### Measurement of endogenous dopamine levels

Approximately 300 to 500 frozen adult hermaphrodites were washed twice with M9 buffer and homogenized in 100 μl of 0.01 N HCl in the presence of EDTA and sodium metabisulfite. Endogenous dopamine extraction, acylation, and measurement were performed by Dopamine ELISA kit - Research (Immusmol, Pessac, France).

#### Movement activity

Movement activities of adult hermaphrodites cultured in each condition was measured by thrashing assay (moving frequency: Hz) and maximal bending angle (degree) described, respectively, with slight modification (Angstman et al., 2016; Ellwood et al., 2021).The frequency for 10 sec of an individual that swims vigourously by gently pushing from outside of the culture bag. The maximal bending angle is defined as the angle between the midpoint-head and midpoint-tail segments, with a straight animal set as 0 degrees (Angstman et al., 2016). For a particular animal, we measured the bending angle of each tracked frame. Live image and muscular cytoplasmic Ca^2+^ levels of adult hermaphrodites in the culture bags were captured by a stereomicroscope (SMZ18, Nikon) and a digital camera (DP73, Olympus) system. Image J software was used to measure the strongest cytoplasmic Ca^2+^ intensity during flexion of the body wall muscles of an individual (n = 16 animals per condition), focused on time-lapse microscopic images with CellSens standard software (Olympus).

#### Dopamine neuron observation and body length measurement

Body length and dopamine neuron morphology with *Pdat-1::GFP* were observed by scanning confocal microscopy (FV10i-ASW, Olympus) immediately after fixation with 100 mM NaN3 solution. Thirty worms were randomly selected for each condition. Number of beads /bubbles in CEP and ADE dopamine neuronal dendrites were counted in D1 (day 1) adults and D4 (day 4) adults.

### Quantification and statistical analysis

Statistical analyses were performed using unequal variance t-test or one-way ANOVA followed by Tukey post hoc tests in RStudio software. P values less than 0.05 were classed as statistically significant. The same alphabet letters in any two groups indicate no significant difference between these groups.

## Data Availability

• All global gene expression data of independent spaceflight experiments are MIAME (Minimum Information about a Microarray Experiment) compliant and are deposited in the GEO (Gene Expression Omnibus) database as the accession numbers listed in the key resources table. • Any additional information required to reanalyze the data reported in this paper is available from the lead contact upon request.
