# Supplementary material for: Loss of physical contact in space alters the dopamine system in C. elegans
Source: iScience. 2022 Jan 11;25(2):103762. doi: 10.1016/j.isci.2022.103762 (PMC8810405; doi:10.1016/j.isci.2022.103762)
Supplement: Document S1. Figures S1–S4 [file mmc1.pdf]

iScience, Volume *25*

Supplemental information

Loss of physical contact in space alters the dopamine system in *C. elegans*

Surabhi Sudevan, Kasumi Muto, Nahoko Higashitani, Toko Hashizume, Akira Higashibata, Rebecca A. Ellwood, Colleen S. Deane, Mizanur Rahman, Siva A. Vanapalli, Timothy Etheridge, Nathaniel J. Szewczyk, and Atsushi Higashitani

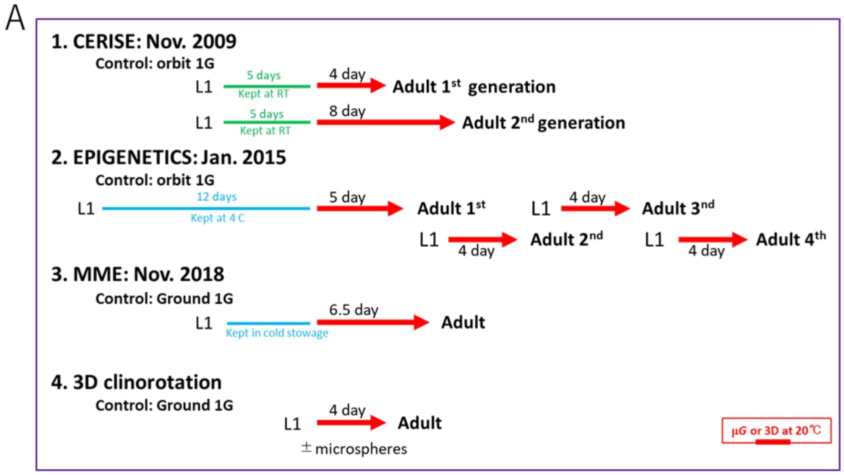

B

Spaceflight samples	*eftu-2/eft-1* FC (μG/1G)	*comt-4* FC (μG/1G)
CERISE 1st	0.88	0.25
CERISE 2nd	0.96	0.33
EPI N2 1st	0.87	0.33
EPI N2 2nd	1.17	0.51
EPI N2 3rd	0.96	0.30
EPI N2 4th	1.10	0.41
EPI hda-4 1st	1.03	0.12
EPI hda-4 2nd	1.27	0.19
EPI hda-4 3rd	1.37	0.17
EPI hda-4 4th	1.25	0.18
EPI sir-2.1 1st	1.17	0.11
EPI sir-2.1 2nd	1.18	0.29
EPI sir-2.1 3rd	1.03	0.25
EPI sir-2.1 4th	0.98	0.21
MME PD55	0.80	0.47

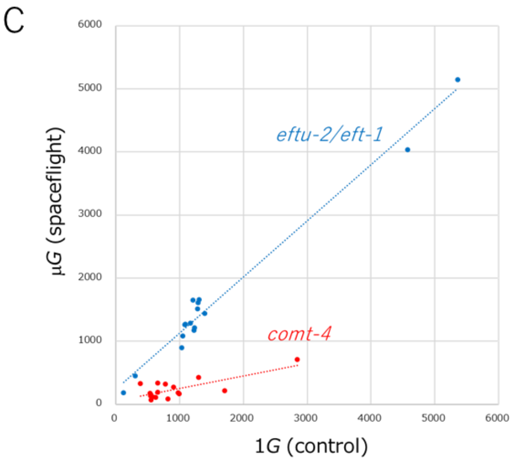

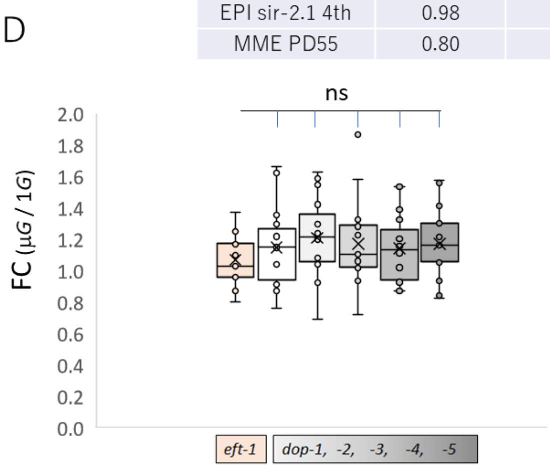

Figure S1. Microgravity-induced *comt-4* suppression was consistently observed over multiple separate ISS flight experiments and within various mutants, related to Figure 1.

(A) schematic for each experimental study. **(B)** *eftu-2/eft-1* and *comt-4* expression in different spaceflight experiments (fold change values with DNA microarray analyses). **(C)** *eftu-2/eft-1* and *comt-4* expression in all 15 microgravity and control conditions. **(D)** Relative expression levels of DA receptor genes, *dop-1, -2, -3, -4* and *-5* did not change under microgravity. Statistical analysis was performed in each condition compared with *eft-1* using Student's t-test. ns: not significant.

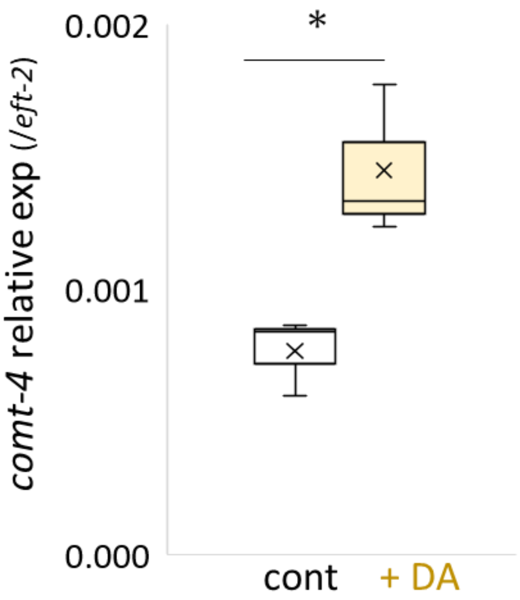

Figure S2. Exogenous dopamine application on Earth upregulates *comt-4* expression analyzed by real-time RT-PCR, related to Figures 1 and 2.

Young adult *C. elegans* (wild-type N2) grown on *E. coli* OP-50 NGM plates were transferred to a freshly prepared OP-50 NGM plate containing a final 50 μM dopamine hydrochloride treatment and further cultured at 20°C for 24 h. Total RNA were extracted from the synchronized adult animals (n = 30) and real-time RT-PCR was performed using *comt-4* and *eef-2* specific primers (Materials and Methods). Data (each biological triplicates) are shown as box and whiskers to indicate median and standard deviations. Statistical analysis was performed in each condition using Student's t-test. * $p < 0.05$.

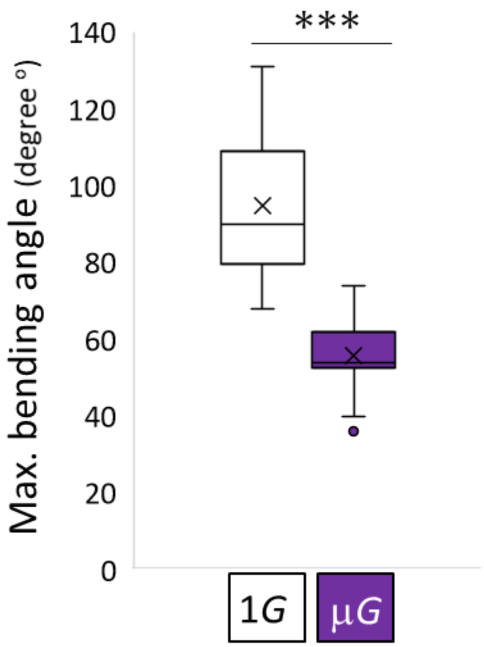

Figure S3. Microgravity reduces maximum animal bending angles, related to Figure 2.

Maximum bending angle (n = 15 adults per condition) were calculated from videos downloaded from the CERISE spaceflight experiment. Data are shown as box and whiskers to indicate median and standard deviations. Statistical analysis was performed in each condition using Student's t-test. *** $p < 0.001$.

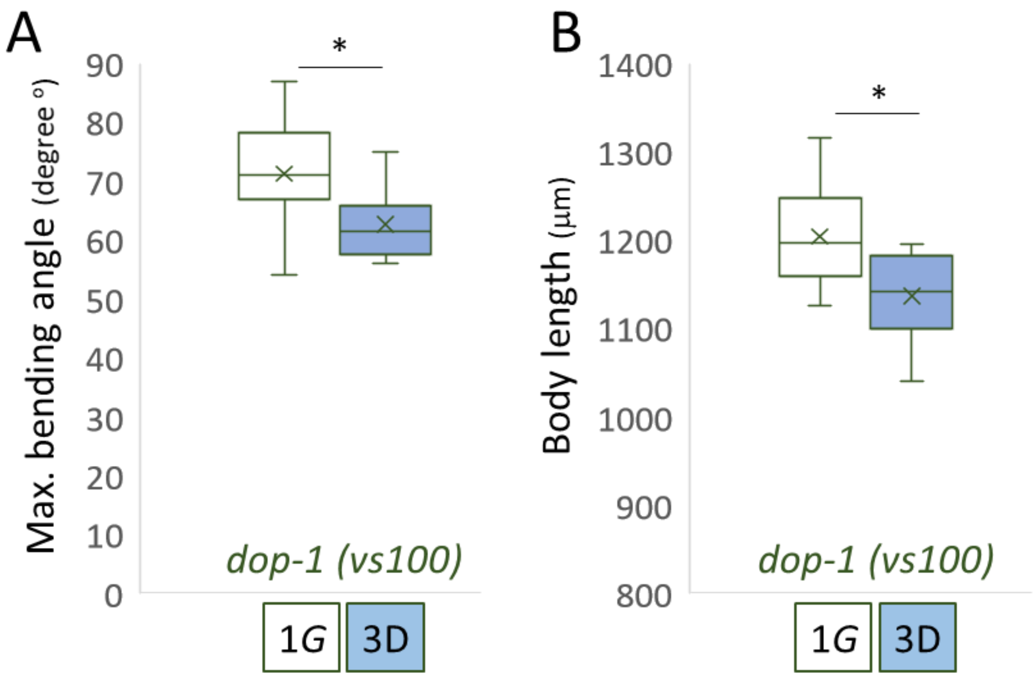

Figure S4. D1-like receptor *dop-1* mutation did not restore moving activity and physique loss under artificial microgravity, related to Figure 3.

(A) Maximal bending angle (n=10 per condition), and **(B)** body length (n=10 per condition) were measured in day 1 adults of N2 and *dop-1* (*vs100*) deletion mutants cultured parallelly under normal gravity (1G) and simulated μG (3D) for 4 days. Data are shown as box and whiskers to indicate median and standard deviations. Statistical analysis was performed in each condition using Student's t-test. * $p < 0.05$.
